# Distribution of Schistosomiasis and Soil Transmitted Helminthiasis in Zimbabwe: Towards a National Plan of Action for Control and Elimination

**DOI:** 10.1371/journal.pntd.0003014

**Published:** 2014-08-14

**Authors:** Nicholas Midzi, Takafira Mduluza, Moses J. Chimbari, Clement Tshuma, Lincoln Charimari, Gibson Mhlanga, Portia Manangazira, Shungu M. Munyati, Isaac Phiri, Susan L. Mutambu, Stanley S. Midzi, Anastancia Ncube, Lawrence P. Muranzi, Simbarashe Rusakaniko, Francisca Mutapi

**Affiliations:** 1 Department of Medical Microbiology, University of Zimbabwe, Harare, Zimbabwe; 2 Department of Biochemistry, University of Zimbabwe, Harare, Zimbabwe; 3 College of Health Sciences, University of KwaZulu Natal, Durban, South Africa; 4 Epidemiology & Disease Control, Ministry of Health and Child Care, Harare, Zimbabwe; 5 Afro-Regional Zimbabwe Offices, World Health Organization, Harare, Zimbabwe; 6 Biomedical Research and Training Institute, Harare, Zimbabwe; 7 National Institute of Health Research, Harare, Zimbabwe; 8 Ministry of Education Sport Arts and Culture, Harare, Zimbabwe; 9 Department of Community Medicine, University of Zimbabwe, Harare, Zimbabwe; 10 University of Edinburgh, Edinburgh, United Kingdom; Ministry of Health, Uganda

## Abstract

**Background:**

Schistosomiasis and STH are among the list of neglected tropical diseases considered for control by the WHO. Although both diseases are endemic in Zimbabwe, no nationwide control interventions have been implemented. For this reason in 2009 the Zimbabwe Ministry of Health and Child Care included the two diseases in the 2009–2013 National Health Strategy highlighting the importance of understanding the distribution and burden of the diseases as a prerequisite for elimination interventions. It is against this background that a national survey was conducted.

**Methodology:**

A countrywide cross-sectional survey was carried out in 280 primary schools in 68 districts between September 2010 and August 2011. *Schistosoma haematobium* was diagnosed using the urine filtration technique. *Schistosoma mansoni* and STH (hookworms, *Trichuris trichiura, Ascaris lumbricoides*) were diagnosed using both the Kato Katz and formol ether concentration techniques.

**Main findings:**

Schistosomiasis was more prevalent country-wide (22.7%) than STH (5.5%). The prevalence of *S. haematobium* was 18.0% while that of *S. mansoni* was 7.2%. Hookworms were the most common STH with a prevalence of 3.2% followed by *A. lumbricoides* and *T. trichiura* with prevalence of 2.5% and 0.1%, respectively. The prevalence of heavy infection intensity as defined by WHO for any schistosome species was 5.8% (range 0%–18.3% in districts). Only light to moderate infection intensities were observed for STH species. The distribution of schistosomiasis and STH varied significantly between provinces, districts and schools (p<0.001). Overall, the prevalence of co-infection with schistosomiasis and STH was 1.5%. The actual co-endemicity of schistosomiasis and STH was observed in 43 (63.2%) of the 68 districts screened.

**Conclusion and recommendations:**

This study provided comprehensive baseline data on the distribution of schistosomiasis and STH that formed the basis for initiating a national control and elimination programme for these two neglected tropical diseases in Zimbabwe.

## Author Summary

Schistosomiasis (*S. haematobium* and *S. mansoni*) and soil transmitted helminthiasis (STH) are endemic in Zimbabwe but there has not been any study conducted to systematically map out the disease for control. Due to the public health significance of schistosomiasis and STH in Zimbabwe, a nationwide cross-sectional survey was conducted to map schistosomiasis and STH in 2010 and 2011, among 13,195 primary school children in 280 primary schools from 68 rural and urban districts in Zimbabwe. Urine filtration technique was used to determine the prevalence and intensity of *S. haematobium*. Both the Kato Katz and formol ether concentration techniques were used to determine the intensity and prevalence of *S. mansoni* and STH (hookworm, *A. lumbricoides* and *Trichuris trichiura*). Overall, the prevalence of schistosomiasis was 22.7% (ranging from 0%–62% in districts) and that of STH was 5.5%, ranging from 0%–45% in districts. There was an overlap of schistosomiasis and STH in space (43 of the 68 districts). Based on the results of this study that confirm nationwide predominance in distribution of schistosomiasis and a lesser extent of STH, Preventive Chemotherapy strategies were determined and recommended for the elimination of these two specific NTD in Zimbabwe.

## Introduction

Schistosomiasis and soil transmitted helminthiasis (STH) are among the most widely distributed neglected tropical diseases (NTDs) that affect people living in vulnerable communities with poor and limited access to safe water, sanitary facilities and inadequate health facilities [1], [2], [3]. Worldwide, over 200 million people are infected with schistosomiasis [1] and more than 1 billion are infected with STH [2]. Morbidity due to schistosomiasis and STH include impairment of cognitive development in young children resulting in poor educational outcome [4], [5], [6], [7]. Soil transmitted helminths also cause anaemia due to worm induced blood loss and compromised nutrition [8], [9], intestinal obstruction as well as reduced absorption of vitamin A, impacting on growth [10], [11]. Of major concern, schistosomiasis has been shown to be a pre-disposing factor for HIV infection [12]. Furthermore, iron deficiency anaemia is exacerbated by co-infection with schistosomiasis, STH and *Plasmodium* malaria [13]. Schistosomiasis and STH may cause reduced birth weight, worker productivity and poor socio-economic development [9]. Thus, control of the two diseases will provide an ancillary contribution towards achieving some Millennium Development Goals; namely, poverty alleviation, universal primary education, reduced child mortality and improved maternal health [14].

A renewed interest for the integrated control of schistosomiasis, STH and other NTDs emerged following the 54.19^th^ World Health Assembly resolution that specified the need to give treatment to at least 75% or all primary school age children at risk of morbidity due to schistosomiasis and STHs by the year 2010 [9], [15], [16], [17]. In 2006 the World Health Organisation introduced the preventive chemotherapy (PCT) strategy encouraging integrated control of overlapping neglected tropical diseases in co-endemic communities. For that to be achieved, data on the extent of overlap in distribution and morbidity levels of the targeted NTDs is essential as it justifies appropriate intervention.

Pursuant to the World Health Organisation's launch of the 2020 Roadmap for implementation of NTD intervention strategies with specific goals for control and elimination, the NTD control initiative received the first ever collaborative donor commitment. Merck KGaA committed to donating 250 million praziquantel tablets annually for an indefinite period towards the treatment of African school children for schistosomiasis. GlaxoSmithKline committed itself to donating up to 400 million tablets per year for STH treatment of school-age children world-wide [14]. The London declaration on NTDs of 30 January 2012 resulted in the largest coordinated action to address NTDs in which partners pledged new levels of collaboration including funding for mapping, tracking and reporting of progress on NTD control (the London Declaration 2012). Member states have expressed political support to the initiative through (i) appointment of steering committees for NTD control and elimination (ii) appointment of the National Focal Persons responsible for spearheading NTD mapping and integrated control in endemic countries [18].

In Zimbabwe, two major national surveys were conducted between 1985 and 1992 [19], [20]. In addition numerous small-scale studies have shown distribution of schistosomiasis in Zimbabwe [21], . In 2012, Chimbari highlighted major pilot studies that were aimed at controlling schistosomiasis in various parts of Zimbabwe. In contrast studies demonstrating the existence of STH were conducted in few loci, namely Burma Valley farming areas and Kariba [23], [24], [25], [26], [27]. Given that Zimbabwe experienced major environmental and socio-economic changes in the past decade and that previous national surveys focused on schistosomiasis in rural areas, the need for a survey mapping both schistosomiasis and STH throughout the country is necessary in order to plan appropriate integrated control strategies [28]. We conducted a national survey to map the current distribution of schistosomiasis and for the first time, to map the distribution of STH in Zimbabwe. Furthermore, we determined current morbidity levels for both infections in order to provide information required for an integrated control program.

## Materials and Methods

### Study design

A school based cross sectional survey was conducted nationwide in rural based provinces between September-October 2010; in metropolitan provinces (Harare and Bulawayo) and Chitungwiza town from July - August 2011. The extension of the national survey from 2010 to 2011 was due to limited financial resources for the project in 2010. The survey was a joint collaboration between the Ministry of Health and Child Care (MOHCC) and the Ministry of Education Sport Arts and Culture (MOESC).

Zimbabwe is divided into 58 administrative districts many of which are rural. Although districts in metropolitan provinces are not recognized by the MOHCC, the MOESC recognises 6 districts in Harare and 5 districts in Bulawayo. For the purpose of this study districts in Harare and Bulawayo were considered, and Chitungwiza, a third largest urban area was divided into two districts (Seke-Chitungwiza and Zengeza) giving a total of 71 districts. Of the 71 districts, the national survey was conducted in 68 (95.8%) as well as peri-urban areas surrounding Harare and Bulawayo. Only three districts (Gweru, Kwekwe and Bindura) were left out due to limited resources.

Ten teams, each made up of two laboratory technicians, one technical assistant, a District Community Nurse, one District Education Officer, the District Environmental Health Officer and a driver conducted data collection. One of the two laboratory technicians was drawn from National Institute of Health Research to lead the team with the overall responsibilities of organising and managing field data collection, filing and safe keeping of research results. This technician was also responsible for performing the urine filtration and the Kato Katz techniques. The second technician drawn from the province was responsible for performing the formol ether concentration technique as well as assisting the team leader in executing other duties. The technical assistant helped in processing specimens as well as cleaning filters, templates and sieves for re-use. The community nurse was responsible for treatment of study participants whilst the District Environmental Officer was responsible for ensuring clean environment in school and at every stage during sample collection as well as feeding children during treatment. The District Education Officer was responsible for locating primary schools randomly selected for the national survey, introducing the research team to the school authorities and for the random selection of primary school children enrolling into the study (participants).

Prior to sample collection, data collection teams were trained on data collection methodologies for 5 days to ensure standardization of data collection procedures. Each of the 10 core teams was given a manual for field data collection and was assigned to collect data in one province for 30 days. Prior to the deployment of the data collection teams, the Secretary for Health and Child Care wrote to all provinces and districts informing Provincial and District Health Executives about the school based national schistosomiasis and STH survey as well as requesting for support including transport and treatment logistics in schools. The Secretary for Education Sport Arts and Culture wrote to all provinces and districts informing the Provincial and District Education Executives about the school based national schistosomiasis and STH survey as well as seeking support for the national data collection teams.

### Ethical issues and permission to conduct the study

The proposal to conduct the national schistosomiasis and STH survey was approved by the national ethical review board, the Medical Research Council of Zimbabwe. The ethical approval number for the study MRCZ/A/1207 dated 11^th^ March 2010. The Secretary for Education Sport Arts and Culture also approved the study. Written informed consent was sought from the parents/guardian of the study participants. UNICEF delivered Parental/guardian informed consent forms addressed to each school by the Secretary for Education Sport Arts and Culture throughout the country in advance to allow school heads sufficient time to liaise with parents/guardians for their consent. On the day of sample collection, only the assenting children whose consent forms were signed by their parents/guardians participated in the study. Enrollment into the study was voluntary. Participants were free to withdraw from the study at any time.

### Study population

Primary school children aged 10–15 years were targeted for the study as they constitute the high-risk age group for schistosomiasis and STH in the community [1] and hence are a proxy of the burden of schistosomiasis and STH in the population [29], [30]. There were exceptions in which non-targeted younger and older children were included in the study population to fulfil the required sample size per school (n = 50).

### Sample size calculation and selection of participants

The national sample size was based on the total enrollment of primary school children, n = 2 490 568 (MOESC 2005). A sample of 15 818 children was calculated using EPI Info 6 statistical package (Epi Info version 6, Centers for Disease Control and Prevention, Atlanta, GA 30333) using 37% as the assumed mean prevalence of schistosomiasis [20] and the error margin of 0.75%. The number of schools selected per district was determined by dividing the district sample size calculated proportionally from the national sample size by the number of children that would be screened per school (n = 50).

Simple random sampling was used to select schools per district using the lottery method [29]. This involved listing names of all primary schools in each district on small slips of paper, then after a thorough mixing of the names, five schools per district were selected one by one. At each school 50 children equally distributed by gender were randomly selected using the lottery method [1], [31]. While the primary school children aged 10–15 years constituted the desired sampling frame, children aged 6–9 (n = 598) and some aged >15 years (n = 6) were included in some schools where the number of children aged 10–15 years was less than 50.

### Parasitological diagnosis and treatment

Single urine, about 100 ml and whole stool specimens were collected in 100 ml screw cap plastic specimen bottles from each child between 1000 and 1400 hours, a period when peak egg excretion is expected [32], [33].

*S. mansoni* and STH were diagnosed using a combination of two diagnostic techniques, (i) the Kato Katz technique [34] and (ii) the formol ether concentration technique [35] in order to improve sensitivity for intestinal helminths diagnosis [36]. A single Kato Katz thick smear was prepared from stool given by each individual for the diagnosis of STHs and *S. mansoni*. This involved straining stool through a Kato Katz sieve with a mesh size of 250 µm. The fine stool was filled in a Kato Katz template producing 41.7 mg. Using the measured fine stool, a Kato Katz thick smear was prepared on a slide and this was covered with the cellophane coverslips soaked in 50% glycerine–malachite green. The slide was examined within 60 minutes of preparation in order to detect and count hookworm eggs before they clear. The slide was left to clear for at least 24 hours after which it was re-examined for *S. mansoni* ova. Using a pear-sized stool (about 1 g) from the remaining stool specimen, the formol ether concentration technique was performed [35]. Results from the Kato Katz and the formal ether concentration techniques for each individual were combined as follows: a person was considered negative for each STH species and *S. mansoni* if no ova of these parasites were detected using both techniques. A person was considered positive for STH species or *S. mansoni* if ova were detected by either of the two or both techniques. Urinary schistosomiasis (*S. haematobium*) was diagnosed using the urine filtration technique [37]. The technique involves filtration of 10 ml of a thoroughly mixed urine specimen, through a Nytrile filter (12–14 µm pore size).

Since the formol ether concentration technique is not quantitative infection intensities for *S. mansoni* and STH were estimated from results obtained using the Kato Katz technique only whilst infection intensity for *S. haematobium* was estimated using the urine filtration techniques In order to estimate infection intensities for intestinal worms, slides were examined from the 41.7 mg stool and all eggs counted. The number of eggs counted was multiplied by 24 to obtain the no of eggs per gram of stool. *Schistosoma haematobium* egg intensity was expressed as the number of eggs per 10 ml urine. The infection intensities of schistosomes and STH species were classified as light, moderate or heavy according to the World Health Organisation thresholds [1]. Using the stratified infection intensities, the prevalence of heavy infection by any schistosome species (morbidity) was expressed as the number of subjects heavily infected with any schistosome species divided by the number of subjects investigated [38]. Sixty-eight districts included in the national survey were classified into different classes of morbidity. Schistosomiasis intervention strategies for districts in each class were proposed based on WHO guidelines for the elimination of schistosomiasis [38]. Due to the low prevalence and light to moderate infection intensities observed, the prevalence of heavy infection with any STH species was not calculated in this study.

In Zimbabwe, a district is an implementation unit. The observed combined prevalence of schistosomiasis was used to classify implementation units (districts) into treatment strategies based on the World Health Organisation prevalence thresholds as follows: high risk area (prevalence ≥50%); moderate risk area (prevalence ≥10% and <50%) and low risk area (prevalence >1% and <10%) [38]. The combined prevalence of STH was also used to stratify implementation units into treatment strategies as follows: high-risk area (prevalence ≥50%) and low risk area (<15%). Due to low prevalence of STH in Zimbabwe the threshold for the low prevalence was reduced from <20% recommended by the World Health Organisation [38] to 15%.

### Co-infections

The prevalence of schistosomiasis -STH co-infections was also calculated. In this study individual schistosomiasis-STHs co-infection was considered for individuals who were infected with at least one schistosome species and at least one soil transmitted helminths species. District *S. haematobium* and *S. mansoni* co-endemicity was defined as the co-existence of *S. haematobium* and *S. mansoni* in the same implementation unit whether due to individuals being co-infected or due to different individuals being infected with different schistosome species District Schistosomiasis -STH co-endemicity was defined as the existence of at least any schistosome species and at least one STH species in the same district whether due to individuals being co-infected or due to different individuals being infected with different helminths.

### Treatment

Following submission of stool and urine samples all participants received orange juice and bread to eat after which they simultaneously received a single dose of praziquantel and albendazole in tablet form at the recommended doses of 40 mg/kg body weight and 400 mg respectively regardless of their infection status since both drugs are considered safe [30].

### Data analysis

Data collected from the field was entered and analysed using the Statistical Package for Social Scientists (SPSS) version 8.0 (SPSS Inc, Chicago, IL, USA). Differences in prevalence of infection among different groups were tested for statistical significance using the Chi-square test. The student's t-test was used to determine the difference in mean age between males and females. The significance level was set at a p-value of 0.05.

Geographical positions of study schools were used to produce maps using geographical information system (GIS) software. The GIS data codification and cleaning was carried out using Microsoft Excel. The clean and coded data were imported into a Microsoft Access database. Further analysis was conducted with Microsoft Access to integrate the different datasets into a single dataset. Then the integrated table was re-imported into Excel sheet before its conversion into the GIS data format. MapInfo (Pitney Bowes Software Inc.) release 6.5 was used to process the data. A GIS data table was created for the schools using their geographic coordinates, which were converted into decimal format from their initial GPS reading format. Out of 280 included in the national survey, 256 (91.4%) had geographic coordinates and were successfully integrated in the school GIS database. The prevalence class breaks are the recommended categorisation by World Health Organization [30].

## Results

A total of 13 195 primary school children drawn from 280 schools were included in the study. Of these, 13067 (99.0%) had their age recoded. Five hundred and ninety seven [597 (4.6%)], 12 464 (95.4%) and 6 (0.05%) were aged 6–9, 10–15 and >15 years old respectively. Ages of 128 participants were not recorded. The mean age (standard deviation) of participants whose ages were recorded was 11.20±1.39 years with males 11.33±1.45 years being significantly older than females 11.07±1.31 years (p<0.0001).

Thirteen thousand and thirty eight (98.8%) of the children examined were screened for *S. haematobium*, 12 249 (92.8%) were screened for *S. mansoni* and 12 252 (92.9%) were screened for STH. Not all participants were screened for all parasites because some could not provide stool or urine specimens on the day of sample collection or had their results missing before entry.

### Prevalence of combined schistosomiasis and STH

Of the 280 schools included in the national survey, schistosomiasis was observed in 237 (84.6%) schools. In schools where schistosomiasis was detected, 116 (48.9%) had moderate prevalence (≥10% but <50%) and 46 (19.5%) had high prevalence (≥50%). Table 1 shows the overall combined prevalence of schistosomiasis: *S. haematobium* and *S. mansoni*; and the overall combined prevalence of STH species: hookworms, *A. lumbricoides* and *T. trichiura* stratified by gender and province. The overall combined prevalence of schistosomiasis was 22.7% ranging from 3.3% to 39.3% in provinces, 0% to 62% in districts and from 0% to 83.7% in schools respectively. Significant variations in prevalence of schistosomiasis between provinces, districts and schools were observed (p<0.0001). The prevalence of combined schistosomiasis was significantly higher in males (25.4%) than in females (20.0%), (p<0.0001).

**Table 1:** Prevalence of combined schistosomiasis and STH infection by province in Zimbabwe in 2011.

Prevalence category	Prevalence of combined schistosomiasis (95%CI)n	Prevalence of combined STH (95%CI)n
**Overall**	22.7 (21.95–23.38)13165	5.5 (5.13–5.94) 12252
**By gender**		
Males	25.4 (24.37–26.50) 6482	5.8 (5.20–6.40) 6042
Females	20.0 (19.0–20.96) 6683	5.3 (4.74–5.78) 6210
**Rural based Province**		
Manicaland	23.8 (22.01–25.75) 2051	4.4 (3.54–5.40) 1978
Mashonaland East	31.2 (28.76–33.71) 1388	18.3 (16.19–20.52) 1269
Mashonaland Central	39.3 (36.32–42.35) 1038	1.8 (1.05–2.78) 1018
Mashonaland West	22.8 (20.54–25.21) 1280	2.4 (1.64–3.44) 1238
Masvingo	37.0 (34.48–38.69) 2054	6.0 (5.01–7.15) 1995
Matabeleland North	3.8 (2.69–5.11) 1037	14.1 (11.93–16.42) 967
Matabeleland South	8.8 (7.09–10.80) 953	0.0 (0.0–0.0) 881
Midlands	30.4 (27.67–33.31) 1058	2.8 (1.81–4.09) 896
**Urban Based (metropolitan) Provinces**		
Harare	9.6 (7.98–11.45) 1165	1.9 (1.17–3.01) 980
Bulawayo	3.3 (2.24–4.75) 871	0.5 (0.13–1.25) 815
Chitungwiza*	5.2 (2.86–8.55) 270	2.8 (1.03–5.97) 215

*  =  Chitungwiza is not a metropolitan province but a town.

Schistosomiasis was predominantly distributed in the north, north-east and eastern regions extending through the central plateaux and the eastern highlands to the south east. The prevalence of schistosomiasis was low in the entire western region of the country. Of the 12 252 participants screened for any STH, the overall combined prevalence of STH was 5.5%, ranging from 0% to 18.3% in provinces, 0% to 45% in districts and 0% to 78.7% in schools. There was no significant difference in prevalence of STH between males (7.5%) and females (6.9%), p = 0.231. The prevalence of STH was highest in Binga district (45.5%, 95%CI = 38.46–52.67) followed by Mutoko (43.5%, 95%CI = 35.55–51.72) and Murehwa district (40.6%, 95%CI = 34.07–47.46). There was significant variation in prevalence of STH between provinces, districts and schools (p<0.0001). Overall, STH were predominantly distributed in the north, north east; eastern region and scantly distributed in the western region of Zimbabwe.

### Prevalence of combined schistosomiasis and STH by settlement type

**Figure 1:**
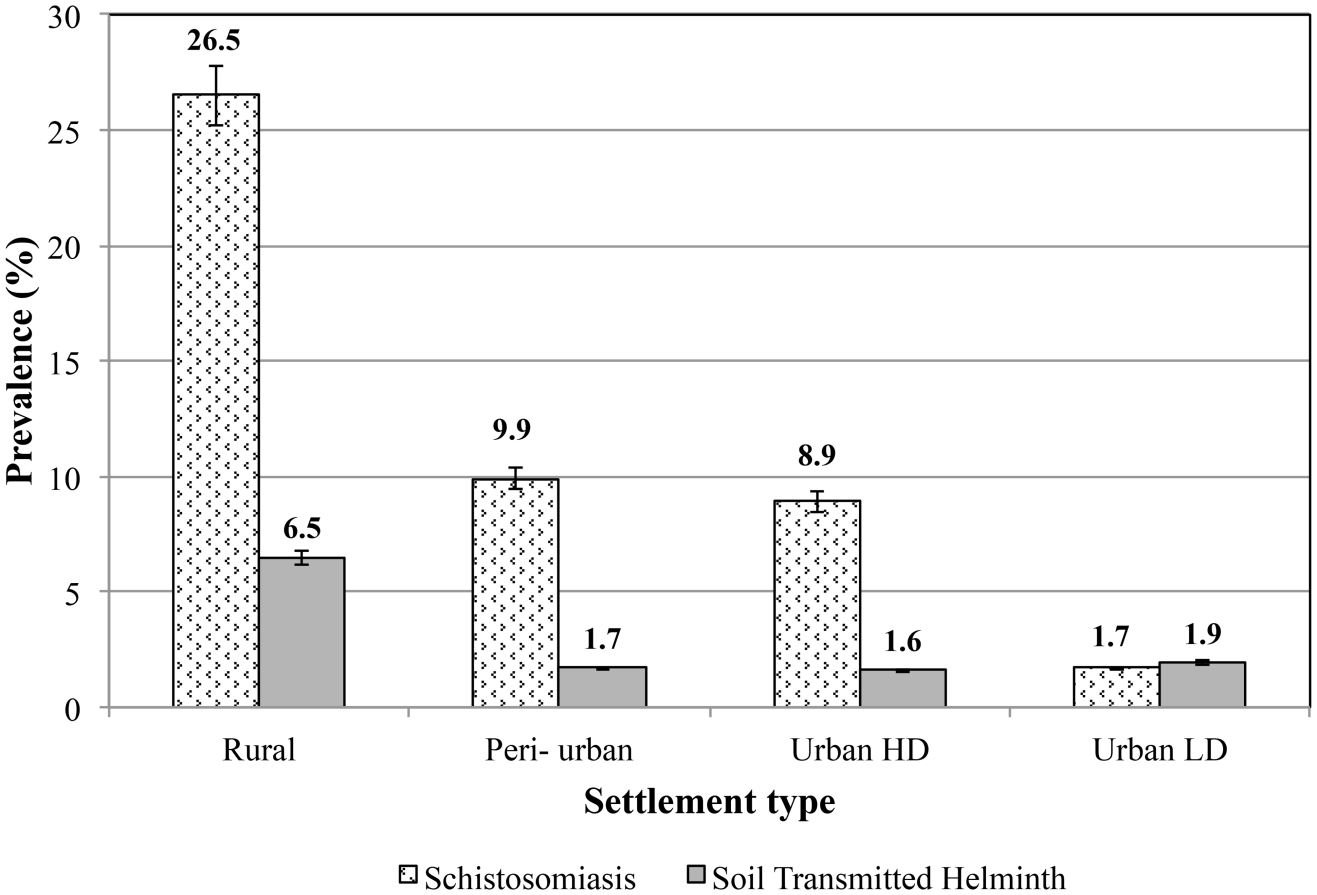
Prevalence distribution of schistosomiasis and STH among 13, 038 primary school children by settlement in Zimbabwe during 2011 school calendar. (HD  =  High density; LD  =  Low density)

Of the 13165 children screened for schistosomiasis (either *S. haematobium* or *S. mansoni*), 10 389 (78.9%) lived in rural areas, 886 (6.7%) lived in peri-urban areas,

12.2% (1 061) lived in urban high-density areas and 2.2% (289) lived in urban-low density areas. Figure 1 describes the prevalence of schistosomiasis by type of settlement. There was a significant variation in prevalence of schistosomiasis by settlement type: rural area (26.5%, 95%CI = 25.60–27.31); peri-urban area (9.9%, 95%CI = 8.04–12.09), urban high-density areas (8.9%, 95%CI = 7.52–10.37) and urban-low density areas (1.7%, 95%CI = 0.56–3.99), p<0.0001.

Of the 12 252 children screened for any STH, 9 828 (80.2%) lived in the rural areas, 830 (6.8%) lived in peri-urban areas, 1 380 (11.3%) lived in urban high-density areas and 214 (1.8%) lived in urban low-density areas. There was a significant variation in prevalence of STH between settlement types (p<0.0001). The prevalence of STH was 6.5% (95%CI = 6.00–6.99) in rural areas; (1.7%, 95%CI = 0.92–2.81) in peri-urban areas; (1.6%, 95% = 1.00–2.40) urban high-density areas and (1.9, 95%CI = 0.50–4.67) urban-low density areas, p<0.0001.

### Prevalence of Schistosome and STH species

**Figure 2:**
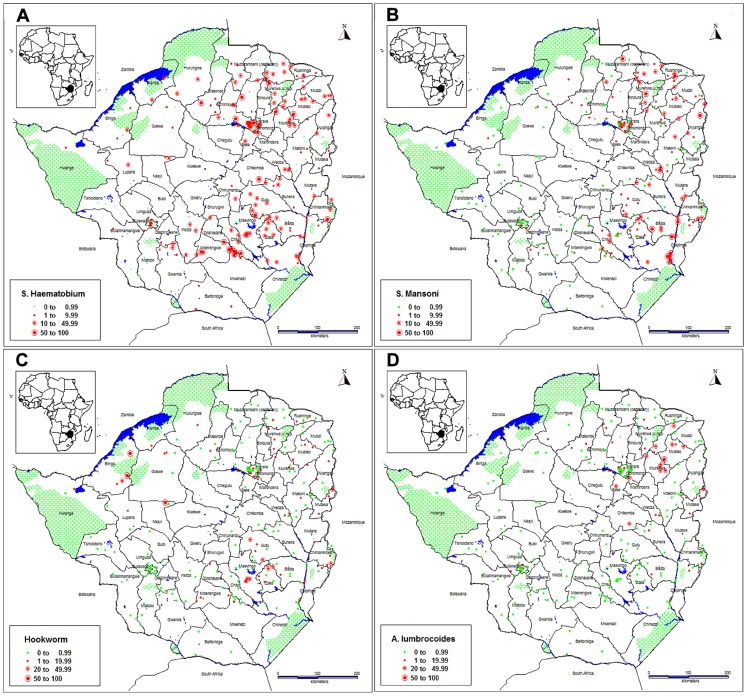
(a) Point prevalence of *S. haematobium* in 280 primary schools in Zimbabwe during 2011 school calendar. (**b**) Point prevalence of *S. mansoni* in 256 primary schools in Zimbabwe during 2011 school calendar. (**c**) Point prevalence of hookworms in 256 primary schools in Zimbabwe during 2011 school calendar. (**d**) Point prevalence of *A. lumbricoides* in 256 primary schools in Zimbabwe during 2011 school calendar.

Table 2 describes the prevalence of different species of schistosomes and STH. Overall, the prevalence of *S. haematobium* was 18.0% (95%CI = 17.38–18.71), ranging from 3.2% to 30.5% in the provinces, 0% to 55.9% in districts and 0% to 76.0% in schools. The prevalence of *S. haematobium* was significantly higher in males (20.8%) than in females (15.4%), p<0.0001. There was a significant variation in prevalence of *S. haematobium* by settlement type: 20.6% (95%CI = 19.87–21.44) in rural areas; 9.8% (95%CI = 7.93–12.02) in peri-urban areas, 8.6% (95%CI = 7.27–10.09) in urban –high density areas and 1.7% (95%CI = 0.57–4.00) in urban-low density areas, p<0.0001. Figure 2a describes the point prevalence of *S. haematobium* indicating its predominance in the country compared with *S. mansoni* that is discontinuously distributed. *Schistosoma haematobium* is highly endemic in the central plateaux, north, north east and the eastern region extending to the south and south east.

**Table 2:** Prevalence of schistosome and STH species by province in Zimbabwe in 2011.

Prevalence Category	*S. haematobium*	*S. Mansoni*	Hookworm	*A. lumbricoides*	*T. trichiura*
Overall prevalence (95%CI)n	18.0 (17.38–18.71) 13037	7.2 (6.74–7.77) 12249	3.2 (2.91–3.54) 12252	2.5 (2.20–2.76)	0.1 (0.07–2.12)
**By gender**					
Males	20.8 (19.80–21.80) 6417	7.5 (8.82–8.16) 6040	3.4 (3.00–3.90) 6042	2.4 (2.06–2.85)	0.2 (0.1–0.34)
Females	15.4 (14.52–16.27) 6620	6.9 (6.31–7.59) 6209	3.0 (2.62–3.48) 6210	2.5 (2.12–2.92)	0.01 (0.02–0.16)
**Rural based Province**					
Manicaland	12.8 (11.33–14.30) 2006	14.3 (12.79–15.93) 1978	2.9 (2.19–3.72) 1978*	1.9 (1.32–2.37) *	0.4 (0.17–0.80)*
Mashonaland East	28.1 (25.72–30.54) 1379	6.4 (5.11–7.88) 1268	1.0 (0.55–1.75) 1269	17.8 (15.74–20.03)	0.2 (0.02–0.57)
Mashonaland Central	26.1 (23.46–28.90) 1034	20.4 (18.00–23.04) 1018	0.6 (0.68–0.22) 1018	1.0 (0.47–1.80)	0.4 (0.11–1.00)
Mashonaland West	22.6 (20.35–20.05) 1259	1.1 (0.56–1.79) 1237	1.6 (0.99–2.48) 1238	1.1 (0.56–1.79)	0.0
Masvingo	27.6 (25.68–29.59) 2054	13.9 (13.40–15.48) 1995	6.0 (5.00–7.10) 1995	0.1 (0.01–0.36)	0.1 (0.01–0.36)
Matabeleland North	3.3 (2.29–4.57) 1032	0.5 (0.17–1.20) 967	14.1 (11.93–16.41) 967	(0.0)*	0.0
Matabeleland South	8.7 (6.95–10.65) 946	0.2 (0.03–0.82) 881	(0.0)** 881	(0.0)*	0.0
Midlands	30.5 (27.76–33.42) 1048	0.3 (0.07–0.97) 896	2.7 (1.72–3.96) 896	0.2 (0.03–0.80)	0.0
**Urban Based (metropolitan) Provinces**					
Harare	9.6 (7.97–11.46) 1154	0.3 (0.06–0.89) 979	1.5 (0.86–2.51) 980	0.5 (0.17–1.29)	0.0
Bulawayo	3.2 (2.09–4.56) 856	0.6 (0.20–1.43) 815	0.1 (0.00–0.68) 815	0.4 (0.08–1.07)	0.0
Chitungwiza	4.8 (2.60–8.12) 269	0.5 (0.01–2.56) 215	1.4 (0.29–4.02) 215	1.9 (0.51–4.69)	0.0

*  =  For each province, the number of participants screened for hookworms, *A. lumbricoides* and *T. trichiura* was the same.

**  =  The prevalence of parasite species was 0%, 95%CI could therefore not be calculated.

The overall prevalence of *S. mansoni* was 7.2% (95%CI = 6.74–7.66) ranging from 0% to 20.4% in provinces, 0% to 43.7% in districts and 0% to 73.6% in schools. Chiredzi district in Masvingo province registered the highest prevalence of *S. mansoni* (43.7%) followed by Hwedza district in Mashonaland East and Nyanga in Manicaland province that had the prevalence of 32.3% and 31.5% respectively. There was no significant difference in prevalence of *S. mansoni* by gender (p>0.231).There was a significant difference in *S. mansoni* prevalence between provinces, districts and schools, p = <0.0001. *Schistosoma mansoni* prevalence varied significantly between settlement types: 8.8% (95%CI = 8.27–9.40) in rural areas; 0.7% (95%CI = 0.35–1.33) in urban high-density areas, 0.5% (95%CI = 0.13–1.23) in peri-urban areas and 0% in urban-low density areas, p>0.0001. Figure 2b describes the point prevalence of *S. mansoni* indicating common occurrence in some parts of the northern region of Zimbabwe (Mashonaland Central province), eastern region (Manicaland province) and in the south east of the country (Masvingo province). It was almost non-existent in the western region of Zimbabwe.

Of the 12 252 participants screened for any STH, the prevalence of hookworm, *A. lumbricoides* and *T. trichiura* was 3.2%; 2.5% and 0.1% respectively (Table 2). Hookworm was the most common STH species occurring in all provinces in Zimbabwe except for Matabeleland South (Table 2 and Figure 2c). Whilst hookworm accounted for the highest prevalence of STH observed in Binga (45.5%), *A. lumbricoides* was predominant in the north-eastern region of the country (Mashonaland East Province) and the Eastern Highlands (Manicaland Province). The point prevalence of *A. lumbricoides* is described in Figure 2d which shows that *A. lumbricoides* was scantly distributed in Zimbabwe and occurred in the north, north-east and Eastern Highlands. *Trichuris trichiura* occurrence is insignificant in Zimbabwe. It was observed in few points in the north-eastern region and the Eastern Highlands of Zimbabwe.

### Infection intensity of schistosomes and STH

The overall arithmetic mean egg intensity for *S. haematobium* and standard error (SE) was 15.0 eggs/10 ml (0.84), range (0–4 000 eggs/10 ml) and that for *S. mansoni* was 7.65 epg (0.67), range (0–4 152 epg). Table 3 describes schistosome infection intensities stratified from light to heavy infection according to the WHO guidelines [1]. The overall prevalence of *S. haematobium* light and heavy infection intensity was 12.4% and 5.6% respectively. The overall prevalence of light, moderate and heavy infection intensity for *S. mansoni* was 3.6%; 1.4% and 0.3% respectively.

**Table 3:** Prevalence of infection intensities of schistosome species and heavy infection with any schistosome species in Zimbabwe stratified by gender and province in 2011.

Prevalence Category	*S. Haematobium* infection intensity		*S. mansoni* infection intensity			Heavy infection with any schistosome species
	Light	Heavy	Light	Moderate	Heavy	% (95% CI)n
Overall prevalence	12.4 (13037)*	5.6	3.6 (12062)*	1.4	0.3	5.8 (5.45–6.26)13160*
**By Gender**						
Males	13.9 (6417)	6.8	3.6 (5951)	1.4	0.3	7.1 (6.46–7.72) 6480
Females	11.0 (6620)	4.4	3.6 (6111)	1.4	0.3	4.7 (4.16–5.19) 6680
**Rural Provinces**						
Manicaland	8.6 (2006)	4.2	8.8 (1939)	0.4	0.3	4.4 (3.55–5.37) 2048
Mashonaland East	19.0 (1378)	9.1	3.7 (1257)	0.8	0.3	9.2 (7.76–10.88) 1386
Mashonaland Central	18.2 (1034)	7.9	8.9 (1016)	4.5	1.3	9.1 (7.38–10.97) 1038
Mashonaland West	16.1 (1259)	6.4	0.3 (1197)	0.0	0.1	6.5 (5.20–7.98) 1280
Masvingo	18.4 (2054)	9.2	5.0 (1916)	1.9	0.6	9.7 (8.49–11.10) 2054
Matabeleland North	2.8 (1032)	0.5	0.2 (965)	0.0	0.3	0.8 (0.33–1.51) 1037
Matabeleland South	6.1 (946)	2.5	0.2 (871)	0.0	0.1	2.6 (1.70–3.85) 953
Midlands	20.8 (1048)	9.7	0.1 (892)	0.0	0.0	9.6 (7.93–11.58) 1058
**Urban Provinces**						
Harare	6.8 (1155)	2.9	1.2 (979)	0.0	0.0	2.9 (2.03–4.05) 1165
Bulawayo	2.8 (856)	0.5	0.6 (815)	0.0	0.0	0.5 (0.13–1.17) 871
Chitungwiza^¤^	3.7 (267)	0.4	0.9 (215)	0.0	0.0	0.4 (0.01–2.05) 270

*  =  Number examined

**¤**  =  Chitungwiza is not a metropolitan province but a town

In order to assess the level of morbidity due to schistosomiasis, the prevalence of heavy infection with any schistosome species was determined and stratified by gender and province (Table 3). Overall, the prevalence of individuals heavily infected with any schistosome species was 5.8%. More males (7.1%) were heavily infected than females (4.7%), p = 0.001. The highest prevalence of heavy infection intensity with any schistosomes species was observed in Masvingo province (9.7%) followed by Midlands (9.6%) and Mashonaland Central province (9.1%) respectively Table 4 classifies the Zimbabwean districts according to the prevalence of heavy infection intensities with any schistosome species (morbidity). Of the 68 districts included in the national schistosomiasis and STH survey, 32 districts (50%) had prevalence of heavy infection with any schistosome species ≥5%. Seventeen districts (25%) had prevalence of heavy infection with any schistosome species ≥1% but <5%. Four districts had the prevalence of heavy infection with any schistosome species >0% but <1% and 15 (22.1%) districts had no detectable morbidity.

**Table 4:** Stratification of 68 districts in Zimbabwe according to prevalence of heavy infection with any schistosome species (morbidity) and the proposed intervention strategies in 2011.

Prevalence category	Districts (IUs)	Comments and intervention strategies
≥10%	Murehwa, Shamva, Mwenezi, Shurugwi, Chikomba, Mutoko, UMP, Hwedza, Mazowe, Mt. Darwin, Zvimba, Chivi, Insiza, Mberengwa (n = 14)	Morbidity is highest, highest transmitting districts. Highest priority requiring uninterrupted intensified PCT with annual geographic coverage of 100% per district. Complementary strategies urgently required. The goal is to control morbidity (reduce prevalence of heavy infection by any schistosome to <5%) in the first 5 years and prevent transmission.
≥5% but <10%	Buhera, Chimanimani, Makoni, Mutare, Mudzi, Seke, Guruve, Muzarabani, Chegutu, Kariba, Kadoma, Chiredzi, Gutu, Masvingo, Zaka, Gwanda, Chirumhanzu, Zvishavane (n = 18)	Morbidity is high. High transmitting districts requiring MDA regularly according to WHO strategies with geographic coverage of 75–100% per district. Complementary strategies are required. The goal is to control morbidity by reducing the prevalence of heavy infection by any schistosome species in the first 5 years to <5% and prevent transmission.
≥1% but <5%	Mutasa, Nyanga, Goromonzi, Marondera, Rushinga, Makonde, Karoyi, Bikita, Hwange, Lupane, Gokwe North, Glenview/Mufakose, Highfields/Glen Norah, Marlbereign/Warren Park, Mabvuku/Tafara, Chitungwiza-Zengeza, Mbare/Hatfield, Khami (n = 17)	Morbidity is moderate though unjustifiable. Moderate transmitting districts. Regular MDA according to WHO guidelines based on prevalence. In addition, identification of transmission foci for intensified PCT is recommended. Complementary strategies are required. The goal is to eliminate schistosomiasis as a public health problem.
<1%	Chipinge, Binga, Beitbridge, Chitungwiza-Seke (n = 4)	Morbidity is low. Low transmitting districts. PCT to be implemented according to WHO guidelines. In addition, monitoring and surveillance of schistosomaisis transmitting foci for intensified PCT is recommended. Complementary strategies are required. The goal is to interrupt transmission.
0%	Bubi, Nkayi, Tsholotsho, Umguza, Bulilima, Matobo, Magwe, Umzingwane, Gokwe South, Reigate, Imbizo, Mzilikazi, Sizinda, North Central (n = 15)	Detailed surveillance should be done to identify any transmitting foci for intensified PCT. Complementary strategies are required. The goal is to interrupt schistosomiasis.

**Key:**

Complementary strategies  =  Health education, safe water and sanitation, environmental management and snail control.

The overall arithmetic mean egg intensity and standard error (SE) for hookworm was 2.99 epg (0.67), range (0–4 488 epg); for *A. lumbricoides* it was 10.97 epg (4.07), range (0–33 912 epg) and 0.25 epg (0.17), range (0–1 416 epg) for *T. trichiura*. The overall prevalence of light infection intensity for hookworm was 0.9%, moderate infection intensity was 0.04%. Only 1 person had heavy infection intensity. The prevalence of light infection intensity for *A. lumbricoides* was 1.0%, moderate infection intensity was 0.03%. Heavy infection intensity with *A. lumbricoides* was not observed. The prevalence of light infection intensity for *T. trichiura* was 0.1% and that for moderate infection intensity was 0.02%. Heavy infection intensity for *T. trichiura* was not observed.

### Schistosomiasis and STH co-infections

Table 5 describes the prevalence of schistosomiasis and STH co-infections by province. Only those participants diagnosed for any or all of the schistosome species and any or all of the STH species (n = 12 257) were considered in this analysis. Overall, 1.5% of the participants had schistosomiasis - STH co-infections, 21.6% had single infection from schistosomiasis and 4.0% had single infection from STH. Schistosomiasis - STH co-infections were observed in 43 (62.3%) of the 68 districts. There was a significant difference in prevalence of schistosomiasis -STH co-infections between provinces and districts with the highest prevalence occurring in Murehwa (19.6%) followed by Mutoko (18.2%) and Masvingo (5.9%) districts respectively, p<0.0001. There was also a significant difference in the prevalence of schistosomiasis-STH co-infections between settlement types with the highest prevalence occurring in the rural areas (1.9%) followed by the peri-urban areas (0.5%), low density urban areas (0.4%) and high density urban areas (0.1%) respectively, p<0.0001.

**Table 5:** Prevalence of schistosomiasis and soil transmitted helminthiasis co-infection combinationsby province in Zimbabwe in 2011.

Category	Number of participants examined	Prevalence of schistosomiasis-STH (95%CI)	Prevalence of schistosomiasis only (95%CI)	Prevalence of STH only (95%CI)
**Overall**	12 257	1.5 (1.32–1.77)	21.6 (20.87–22.33)	4.0 (3.61–4.32)
**Gender**				
Males	6 043	1.6 (1.33–1.99)	24.4 (23.28–25.46)	4.1 (3.62–4.64)
Female	6 214	1,4 (1.18–1.81)	18.9 (17.94–19.91)	3.8 (3.35–4.32)
**Rural based provinces**				
Manicaland	1 977	0.8 (0.43–1.25)	23.1 (21.72–25.04)	3.6 (2.86–4.64)
Mashonaland East	1 271	7.6 (6.16–9.15)	24.7 (22.36–27.17)	10.4 (8.76–12.19)
Mashonaland Central	1 019	1.2 (0.61–2.05)	38.4 (35.37–41.44)	0.5 (0.16–1.14)
Mashonaland West	1 238	0.9 (0.44–1.58)	21.6 (19.38–24.05)	1.5 (0.01–0.02)
Masvingo	1 996	1.8 (1.27–2.49)	35.5 (33.27–37.51)	4.2 (3.37–5.18)
Matabeleland North	967	0.8 (0.36–1.62)	2.9 (1.93–4.16)	13.2 (11.16–15.54)
Matabeleland South	881	0.0	9.0 (7.16–11.05)	0.0
Midlands	897	0.6 (0.18–1.30)	30.5 (27.54–33.67)	2.2 (1.8–3.42)
Harare	982	0.4 (0.11–1.04)	9.4 (7.62–11.37)	1.5 (0.86–2.51)
Bulawayo	814	0.0	3.4 (0.02–0.05)	0.5 (0.00–0.01))
Chitungwiza	215	0.0	3.3 (1.32–6.59)	2.8 (1.03–5.97)

Overall, *S. haematobium* – *S. mansoni* co-infections were observed in 252 (2.1%) of the 12140 participants screened for both schistosomes species. The overlap of *S. haematobium* and *S. mansoni* was predominant in the northern region of the country (Mashonaland central province) in which the prevalence of co-infection was 7.0%. The prevalence of *S. haematobium - S. mansoni* co-infection was 12.8% in two districts, Hwedza which is located in the eastern region of the country (in Mashonaland East province) and Mt. Darwin located to the north of Zimbabwe (in Mashonaland Central province). Chiredzi district is located to the south of Zimbabwe (Masvingo province) had a prevalence of *S. haematobium* and *S. mansoni* co-infection of 10.5%.

### Treatment strategies

Table 6 shows the classification of Zimbabwean districts according to the prevalence and overlap of schistosomiasis and STH observed in this study. The prevalence categories are recommended by WHO and form the basis for which different intervention strategies are specified for the control of schistosomiasis and STH [30]. Of the 68 districts included in the national survey, the prevalence of schistosomiasis ≥50% was observed in 5 rural based districts. Prevalence of schistosomiasis ≥10% but <50% was observed in 39 of the 68 districts (57.4%). Eighteen districts (26.5%) had the prevalence of schistosomiasis >0% but <10%. Seven rural and urban districts had prevalence of 0% whilst peri-urban areas around Harare and Bulawayo had an average schistosomiasis prevalence of 10.7% and 1.9% respectively. Eight districts had the prevalence of STH ≥15% whilst 35 (51.5%) districts had the prevalence of STH >0.0% but <15%. Schistosomiasis was not detected in 9 of the 25 districts that were non-endemic to STH.

**Table 6:** Classification of 68 districts in Zimbabwe according to prevalence and overlap of schistosomiasis and STH in 2011.

Prevalence categories for schistosomiasis and STHs		Districts in the category (n)
Schistosomiasis	STHs	
≥50%	<15%	Hwedza, Shamva, Chiredzi, Shurugwi, Chikomba (n = 5)
≥10% but <50%	≥15%	Murehwa, Mutoko, Seke, UMP, (n = 4)
≥10% but <50%	<15%	Buhera, Chimanimani, Chipinge, Makoni, Mutare, Nyanga, Mudzi, Guruve, Mazowe, Mt. Darwin, Muzarabani, Kadoma, Rushinga, Chegutu, Kariba, Makonde, Zvimba, Bikita, Chivi, Gutu, Masvingo, Mwenezi, Zaka, Insiza, Chirumhanzu, Gokwe North, Mberengwa, Karoyi, Zvishavane, Glenview/Mufakose, Marbereigne/Warren Park, Sizinda, Gwanda (n = 33).
>0.0% but <10%	≥15%	Mutasa, Binga, Nkayi (n = 3)
>0.0% but <10%	<15%	Lupane, Hwange, BeitBridge, Bulilima, Umzingwane, Gokwe South, Highfields/Glen Norah, Mabvuku/Tafara, Mbare/Hatfield, Zengeza, Chitungwiza –Seke, Reigate, Mzilikazi, Khami, Goromonzi, Marondera (n = 16)
0%	0%	Bubi, Tsholotsho, Umguza, Matobo, Magwe, North Central, Imbizo (n = 7)

**Figure 3:**
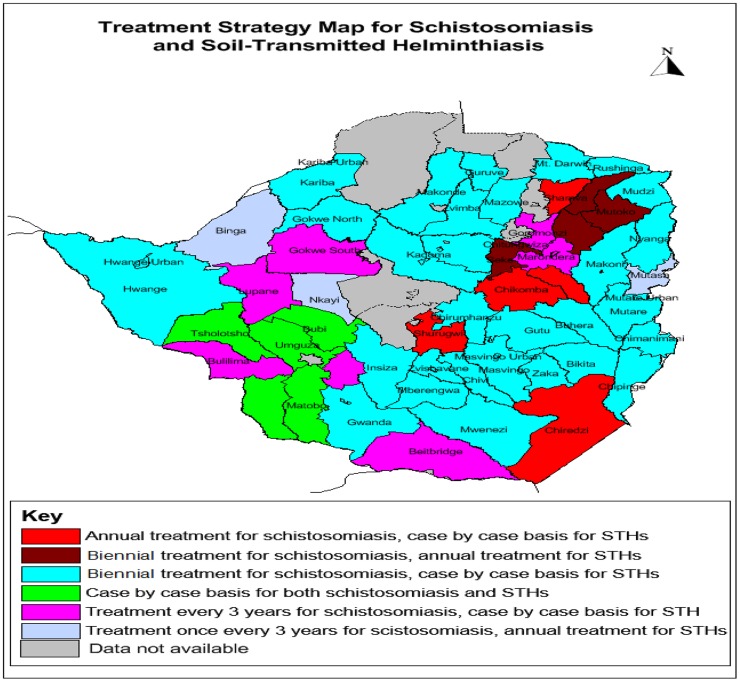
Preventive chemotherapy strategies recommended in 68 districts of Zimbabwe based on schistosomiasis and STH prevalence and co-endemicity in 2011.

Schistosomiasis–STH co-endemicity was observed in 41 of the 68 districts (60.3%). Peculiar was the observed high prevalence of both schistosomiasis and STH in Murehwa: 40.6% and 43.5% respectively, and Mutoko: 47.4% and 36.1% districts respectively. Figure 3 shows the recommended Preventive Chemotherapy (PCT) strategies based on prevalence and schistosomiasis-STH co-infections according to WHO guidelines. On the basis of district level prevalence results (Table 6), annual mass drug administration for the control of schstosomiasiss was predicted in 5 districts red colour) whilst annual mass drug administration (MDA) for the control of STH was predicted in 7 districts. Biennial MDA for schistosomiasis control was predicted in 33 districts (Figure 3, blue and brown colours).

## Discussion

This study is the first to show the national distribution of both schistosomiasis and STH in rural, metropolitan provinces and the surrounding peri-urban areas making it possible to compare the distribution of the two diseases in urban, peri-urban and rural areas in Zimbabwe. Furthermore, we were able to assess the morbidity levels for schistosomiasis and STH which previous surveys [19], did not do. The use of the formol ether concentration technique in this study increased the sensitivity in diagnosis of *S. mansoni* and STH Our results show that, of the 13195 participants screened for *S. mansoni* using both the Kato Katz and formol ether, 641 (5.3%) were diagnosed positive by the earlier technique and 672 (5.5%) were diagnosed positive by the later technique. However, after combining results obtained using the two techniques, 881 (7.2%) were diagnosed positive for *S. mansoni*. These observations are corroborated with results reported by [36].

Single day urine and stool specimens were examined and hence the real prevalence may have been underestimated as daily variability in egg excretion has been reported [33], [39], [40]. The strength of our study is that we have made it possible to classify districts based on what implementation strategy needs to be adopted in line with the WHO recommendations for Preventive Chemotherapy (Figure 3). We have also reported the combined prevalence of schistosomiasis and STH previously unreported for Zimbabwe [19] but which is the operational parameter for control programmes.

The observed national distribution of SCH indicating a lesser problem in the western part of the country [Figure 1(a) and (b)], generally corroborates findings of the previous surveys [19], [20]. While Taylor and Makura in 1985 divided the country into three zones according to the prevalence of schistosomiasis, we were able to determine the prevalence of schistosomiasis at provincial and district levels (Tables 3 and 6, and Figure 3). Given that resources are allocated to provinces and subsequently districts, our results make planning for control interventions more practical.

This study was cross sectional in nature and therefore could not provide empirical data on causality. Thus observations made can only be cautiously explained. The persistence of high prevalence of schistosomiasis throughout periods of all the 3 national surveys [19], [20] and current survey is not surprising as no control measures were instituted following the previous two surveys. Furthermore, the country went through serious social and environmental changes favouring transmission of schistosomiasis in the past two decades [41]. However, the benefits of sporadic localized control interventions are evident in areas where pilot schistosomiasis control projects [42], [43], [44], [45] were implemented. Contrary to the case of schistosomiasis, national distribution of STH was mapped for the first time in Zimbabwe through this study. The results showed that STH tends to occur in areas where schistosomiasis occurs, being common in the north, north eastern region, the eastern highlands and the southern region of Zimbabwe. The observed overlap of STH and schistosomiasis is consistent with observations made by Yajima *et al* in 2011 [46].

The overlap in distribution of schistosomiasis and STH in Zimbabwe may be explained by the overlap of conditions that favour co-existence of both schistosomiasis and STH. Putatively, these include existence of wet soils maintained by high rainfalls, optimal temperatures for parasites survival and livelihoods activities that pre-dispose communities to infections. The livelihoods activities like market gardening in the endemic areas, bathing and washing clothes in rivers increase the frequency of exposure of people to contaminated water and general poor sanitation facilities result in environmental contamination with faeces. The observed high prevalence of schistosomiasis in the rural areas compared to urban and peri-urban areas may be attributed to inadequate sanitary facilities, use of contaminated water sources for domestic chores that include washing clothes and bathing. It could also be due to risky (in terms of predisposition to schistosomiasis infection) livelihoods activities such as market gardening and brick moulding that are inherent in rural Zimbabwe. The situation in peri-urban areas is also worse than those in urban areas for some of the above stated reasons in particular poor sanitation and inadequate safe water for domestic use.

The World Health Organisation set stepwise criteria for morbidity control, schistosomiasis elimination and interruption [47]. According to this criteria, morbidity control can be achieved if the prevalence of heavy infection intensity with any schistosome species in an endemic country is reduced to <5%; elimination of schistosomiasis will be achieved if the prevalence of heavy infection intensity with any schistosome species is reduced to <1% and interruption is achieved by reducing the incidence of infection to zero [47]. Our results show that Zimbabwe has an overall prevalence of heavy infection intensity with any schistosome species of 5.8%; 32 (47.1%) districts had the prevalence of heavy infection intensity with any schistosome species >5% and 51 (75.0%) of the 68 districts included in this study have the prevalence of heavy infection intensity by any schistosome species >1% (Table 4). It should however be noted that as indicated above examination of single urine and stool sample may have underestimated the prevalence and morbidity of infections. Thus, in order for Zimbabwe to achieve the set global goals: to eliminate schistosomiasis by 2020 and interrupt it by 2025 respectively [47], the country should implement uninterrupted PCT and complementary strategies including health education, improved sanitation and safe water supply as recommended by this study. Fourteen districts had the prevalence of heavy infection intensity by any schistosome species >10% (Table 4). These include all those districts classified as high-risk areas based on prevalence of infection except for Chiredzi district (Table 6). It is therefore recommended that all the 14 districts be considered as high risk areas requiring annual mass praziquantel treatment as opposed to 5 districts identified based on prevalence only (Table 6 and Figure 3).

While co-infection of individuals is associated with aggravated co-morbidity and is a cause of global concern [13], [30], results from the present study show low prevalence of individuals with schistosomiasis-STH co-infections (1.5%) even when stratified by province (Table 5). Our findings also show that whilst the prevalence of schistosomiasis –STH co-infection was only 1.5% in individuals, 41 of the 68 districts (60.3%) had schistosomiasis-STH co-endemicity. Thus, STH and schistosomiasis overlap more in areas than in individuals in Zimbabwe. This could be attributed to the low prevalence of STH across the country except for Mashonaland East province where the prevalence of both schistosomiasis and STH is high (Table 1). On the basis of results provided in this study and in order to eliminate preventive chemotherapy NTDs, we suggest that in areas where one NTD is highly endemic, the condition should trigger co-administration of medicines including medicines for the treatment of other NTDs occurring at threshold levels even lower than those specified by WHO for PCT [30]. Using this approach, elimination of STH can naturally be possible in Zimbabwean setting and elsewhere.

### Conclusion and recommendations

The present study provides comprehensive baseline data showing geographic distribution of schistosomiasis and STH, their co-endemicity and the prevalence of heavy infection with any schistosome species in Zimbabwe. Intervention strategies intended to direct the country in controlling morbidity, eliminate and interrupt schistosomiasis and STH transmission are suggested. Uninterrupted annual MDA is recommended in districts where the prevalence of schistosomiasis and STH is ≥50% and ≥15% respectively. Similarly, annual MDA is recommended in districts where the prevalence of heavy infection by any schistosome species is ≥10%. Complementary strategies including health education, provision of safe water and adequate sanitary facilities should concurrently be implemented. The intervals for the co-administration of different medicines directed at different PCT neglected tropical diseases should be dependent on the NTD with the highest prevalence in any implementation unity.

## Supporting Information

Checklist S1 **STROBE checklist.** 1. *(a)* The study design (*b*) An informative and balanced summary of what was done and what was found. 2. The scientific background and rationale for the investigation. 3. Specific objectives, including any pre-specified hypotheses. 4. Key elements of study design. 5. The setting, locations, and dates, periods of recruitment, exposure, follow-up, and data collection. 6. Eligibility criteria, and the sources and methods of selection of participants. 7. Outcomes, exposures, predictors, potential confounders, and effect modifiers. 8. Sources of data and details of methods of assessment (measurement). 9. Potential sources of bias. 10.The study size. 11. Quantitative methods. 12. Statistical methods *(a*) Statistical methods, including those used to control for confounding *(b*) Methods used to examine subgroups and interactions (*c*) Missing data (*d*) Analytical methods taking account of sampling strategy (*e*) Sensitivity analyses. 13. Participants *(a)* Numbers of individuals at each stage of study—e.g. numbers potentially eligible, examined for eligibility, confirmed eligible, included in the study, completing follow-up, and analysed *(b)* Reasons for non-participation. 14. *(a)* Characteristics of study participants (e.g. demographic, clinical, social) and information on exposures and potential confounders *(b)* Number of participants with missing data for each variable of interest. 15. Numbers of outcome events or summary measures. 16. Main results. 17.Other analyses done—e.g. analyses of subgroups and interactions, and sensitivity analyses. 18. Summary of key results with reference to study objectives. 19. Limitations of the study, taking into account sources of potential bias or imprecision. 20. Overall interpretation of results considering objectives, limitations, multiplicity of analyses, results from similar studies, and other relevant evidence. 21. The source of funding and the role of the funders.
(DOCX)
