# Supplementary material for: Distribution of Schistosomiasis and Soil Transmitted Helminthiasis in Zimbabwe: Towards a National Plan of Action for Control and Elimination
Source: PLoS Negl Trop Dis. 2014 Aug 14;8(8):e3014. doi: 10.1371/journal.pntd.0003014 (PMC4133179; doi:10.1371/journal.pntd.0003014)
Supplement: Checklist S1 — STROBE checklist. 1. (a) The study design (b) An informative and balanced summary of what was done and what was found. 2. The scientific background and rationale for the investigation. 3. Specific objectives, including any pre-specified hypotheses. 4. Key elements of study design. 5. The setting, locations, and dates, periods of recruitment, exposure, follow-up, and data collection. 6. Eligibility criteria, and the sources and methods of selection of participants. 7. Outcomes, exposures, predictors, potential confounders, and effect modifiers. 8. Sources of data and details of methods of assessment (measurement). 9. Potential sources of bias. 10.The study size. 11. Quantitative methods. 12. Statistical methods (a) Statistical methods, including those used to control for confounding (b) Methods used to examine subgroups and interactions (c) Missing data (d) Analytical methods taking account of sampling strategy (e) Sensitivity analyses. 13. Participants (a) Numbers of individuals at each stage of study—e.g. numbers potentially eligible, examined for eligibility, confirmed eligible, included in the study, completing follow-up, and analysed (b) Reasons for non-participation. 14. (a) Characteristics of study participants (e.g. demographic, clinical, social) and information on exposures and potential confounders (b) Number of participants with missing data for each variable of interest. 15. Numbers of outcome events or summary measures. 16. Main results. 17.Other analyses done—e.g. analyses of subgroups and interactions, and sensitivity analyses. 18. Summary of key results with reference to study objectives. 19. Limitations of the study, taking into account sources of potential bias or imprecision. 20. Overall interpretation of results considering objectives, limitations, multiplicity of analyses, results from similar studies, and other relevant evidence. 21. The source of funding and the role of the funders. (DOCX) [file pntd.0003014.s001.docx]

STROBE Statement—Checklist of items that should be included in reports of ***cross-sectional studies***

|  | Item No | Recommendation |
| --- | --- | --- |
| **Title and abstract** | 1 | (*a*) A countrywide cross sectional survey |
|  |  | (*b*) Provide in the abstract an informative and balanced summary of what was done and what was found  A countrywide cross sectional survey was carried out in 280 primary schools in 68 districts between September 2010 and August 2011. *Schistosoma haematobium* was diagnosed using the urine filtration technique. *Schistosoma mansoni* and STH (hookworms, *Trichuris trichiura, Ascaris lumbricoides*) were diagnosed using both the Kato Katz and formol ether concentration techniques. Schistosomiasis was more prevalent country-wide (22.7%) than STH (5.5%). The prevalence of *S. haematobium* was 18.0% while that of *S. mansoni* was 7.2%. Hookworms were the most common STH with a prevalence of 3.2% followed by *A. lumbricoides* and *T. trichiura* with prevalence of 2.5% and 0.1%, respectively. The prevalence of heavy infection intensity as defined by WHO for any schistosome species was 5.8% (range 0%-18.3% in districts). Only light to moderate infection intensities were observed for STH species. The distribution of schistosomiasis and STH varied significantly between provinces, districts and schools (p < 0.001). Overall, the prevalence of co-infection with schistosomiasis and STH was 1.5%. The actual co-endemicity of schistosomiasis and STH was observed in 43 (63.2%) of the 68 districts screened. This study provided comprehensive baseline data on the distribution of schistosomiasis and STH that formed the basis for initiating a national control and elimination programme for these two neglected tropical diseases in Zimbabwe. |
| Introduction | | |
| Background/rationale | 2 | Explain the scientific background and rationale for the investigation being reported:  Schistosomiasis and STH are among the list of neglected tropical diseases considered for control by the WHO. Although both diseases are endemic in Zimbabwe, no nationwide control interventions have been implemented. For this reason in 2009 the Zimbabwe Ministry of Health and Child Care included the two diseases in the 2009-2013 National Health Strategy highlighting the importance of understanding the distribution and burden of the diseases as a pre-requisite for elimination interventions. It is against this background that a national survey was conducted. |
| Objectives | 3 | State specific objectives, including any prespecified hypotheses:  Given that Zimbabwe experienced major environmental and socio-economic changes in the past decade and that previous national surveys focused on schistosomiasis in rural areas, the need for a survey mapping both schistosomiasis and STH throughout the country was necessary in order to plan appropriate integrated control strategies. We conducted a national survey to map the current distribution of schistosomiasis and for the first time, to map the distribution of STH in Zimbabwe. Furthermore, we determined current morbidity levels for both infections in order to provide information required for an integrated control program. |
| Methods | | |
| Study design | 4 | Present key elements of study design early in the paper:  A school based cross sectional survey was conducted nationwide in rural and in metropolitan provinces. A total of 13 195 primary school children aged 10-15 years drawn from 280 primary schools. The study population comprised of 50 children per school. |
| Setting | 5 | Describe the setting, locations, and relevant dates, including periods of recruitment, exposure, follow-up, and data collection  A school based cross sectional survey was conducted nationwide in rural based provinces between September-October 2010; in metropolitan provinces (Harare and Bulawayo) and Chitungwiza town from July - August 2011. The extension of the national survey from 2010 to 2011 was due to limited financial resources for the project in 2010. The survey was a joint collaboration between the Ministry of Health and Child Care (MOHCC) and the Ministry of Education Sport Arts and Culture (MOESC). |
| Participants | 6 | Give the eligibility criteria, and the sources and methods of selection of participants  A school based cross sectional survey was conducted nationwide in randomly selected primary schools. School children aged 10-15 years were targeted for the study as they constitute the high-risk age group for schistosomiasis and STH in the community and hence are a proxy of the burden of schistosomiasis and STH in the population. There were exceptions in which non-targeted younger and older children were included in the study population to fulfil the required sample size per school (n = 50). A total of 13 195 primary school children drawn from 280 schools were included in the study. |
| Variables | 7 | Clearly define all outcomes, exposures, predictors, potential confounders, and effect modifiers. Give diagnostic criteria, if applicable  School children were examined for parasitological infection in urine and stool samples. Ova were detected in the infected children. Data collected from the field was entered as egg counts while another variable was considered of either infected or uninfected by show of ova present and analysed. It should however be noted that examination of single urine and stool sample may have underestimated the prevalence and morbidity of infections. *S. mansoni* and STH were diagnosed using a combination of two diagnostic techniques, (i) the Kato Katz technique and (ii) the formol ether concentration technique in order to improve sensitivity for intestinal helminths diagnosis. A single Kato Katz thick smear was prepared from stool given by each individual for the diagnosis of STHs and *S. mansoni* infection intensity. Results from the Kato Katz and the formol ether concentration techniques for each individual were combined as follows: a person was considered negative for each STH species and *S. mansoni* if no ova of these parasites were detected using both techniques. A person was considered positive for STH species or *S. mansoni* if ova were detected by either of the two or both techniques. Urinary schistosomiasis (*S. haematobium*) was diagnosed using the urine filtration technique. The technique involves filtration of 10 ml of a thoroughly mixed urine specimen, through a Nytrile filter (12-14µm pore size). |
| Data sources/ measurement | 8* | For each variable of interest, give sources of data and details of methods of assessment (measurement). Describe comparability of assessment methods if there is more than one group  **Source of data**  Single urine, about 10ml and whole stool specimens were collected in 100 ml screw cap plastic specimen bottles from each child between 1000 and 1400 hours, a period when peak egg excretion is expected. Urinary schistosomiasis (*S. haematobium*) was diagnosed using the urine filtration technique. The technique involves filtration of 10 ml of a thoroughly mixed urine specimen, through a Nytrile filter (12-14µm pore size).  *S. mansoni* and STH were diagnosed using a combination of two diagnostic techniques,  (i) the Kato Katz technique and  (ii) the formol ether concentration technique in order to improve sensitivity for intestinal helminths diagnosis. A single Kato Katz thick smear was prepared from stool given by each individual for the diagnosis of STHs and *S. mansoni*. This involved straining stool through a Kato Katz sieve with a mesh size of 250 µm. The fine stool was filled in a Kato Katz template producing 41.7mg. Using the measured fine stool, a Kato Katz thick smear was prepared on a slide and this was covered with the cellophane coverslips soaked in 50% glycerine-malachite green. The slide was examined within 60 minutes of preparation in order to detect and count hookworm eggs before they clear. The slide was left to clear for at least 24 hours after which it was re-examined for *S. mansoni* ova. Using a pear sized stool (about 1g) from the remaining stool specimen, the formol ether concentration technique was performed. Results from the Kato Katz and the formol ether concentration techniques for each individual were combined as follows: a person was considered negative for each STH species and *S. mansoni* if no ova of these parasites were detected using both techniques. A person was considered positive for STH species or *S. mansoni* if ova were detected by either of the two or both techniques.  Since the formol ether concentration technique is not quantitative infection intensities for *S. mansoni* and STH were estimated from results obtained using the Kato Katz technique only whilst infection intensity for *S. haematobium* was estimated using the urine filtration techniques In order to estimate infection intensities for intestinal worms, slides were examined from the 41.7mg stool and all eggs counted. The number of eggs counted was multiplied by 24 to obtain the no of eggs per gram of stool. *Shstosoma haematobium* egg intensity was expressed as the number of eggs per 10 ml urine. The infection intensities of schistosomes and STH species were classified as light, moderate or heavy according to the World Health Organisation thresholds. Using the stratified infection intensities, the prevalence of heavy infection by any schistosome species (morbidity) was expressed as the number of subjects heavily infected with any schistosome species divided by the number of subjects investigated. Sixty-eight districts included in the national survey were classified into different classes of morbidity. Schistosomiasis intervention strategies for districts in each class were proposed based on WHO guidelines for the elimination of schistosomiasis. Due to the low prevalence and light to moderate infection intensities observed, the prevalence of heavy infection with any STH species was not calculated in this study. |
| Bias | 9 | Describe any efforts to address potential sources of bias  Data collection was conducted by 10 teams, each made up of two laboratory technicians, one technical assistant, a District Community Nurse, one District Education Officer, the District Environmental Health Officer and a driver. One of the two laboratory technicians was drawn from National Institute of Health Research to lead the team with the overall responsibilities of organising and managing field data collection, filing and safe keeping of research results. The District Education Officer was responsible for locating primary schools randomly selected for the national survey, introducing the research team to the school authorities and for the random selection of primary school children enrolling into the study (participants). There were exceptions in which non-targeted younger and older children were included in the study population to fulfil the required sample size per school (n = 50). |
| Study size | 10 | Explain how the study size was arrived at  Using 37% as the assumed mean prevalence of schistosomiasis from previous localized surveys and the error margin of 0.75%. The number of schools selected per district was determined by dividing the district sample size calculated proportionally from the national sample size by the number of children that would be screened per school (n =50). Simple random sampling was used to select schools per district using the lottery method. This involved listing names of all primary schools in each district on small slips of paper, then after a thorough mixing of the names, five schools per district were selected one by one. At each school 50 children equally distributed by gender were randomly selected using the lottery method. While the primary school children aged 10-15 years constituted the desired sampling frame, children aged 6-9 (n = 598) and some aged > 15 years (n = 6) were included in some schools where the number of children aged 10-15 years was less than 50. Zimbabwe is divided into 58 administrative districts many of which are rural. Although districts in metropolitan provinces are not recognized by the MOHCC, the MOESC recognises 6 districts in Harare and 5 districts in Bulawayo. For the purpose of this study districts in Harare and Bulawayo were considered, and Chitungwiza, a third largest urban area was divided into two districts (Seke-Chitungwiza and Zengeza) giving a total of 71 districts. Of the 71 districts, the national survey was conducted in 68 (95.8%) as well as peri-urban areas surrounding Harare and Bulawayo. Only three districts (Gweru, Kwekwe and Bindura) were left out due to limited resources. |
| Quantitative variables | 11 | Explain how quantitative variables were handled in the analyses. If applicable, describe which groupings were chosen and why  A school based cross sectional survey was conducted nationwide in randomly selected primary schools. School children aged 10-15 years were targeted for the study as they constitute the high-risk age group for schistosomiasis and STH in the community and hence are a proxy of the burden of schistosomiasis and STH in the population. There were exceptions in which non-targeted younger and older children were included in the study population to fulfil the required sample size per school (n = 50). A total of 13 195 primary school children drawn from 280 schools were included in the study. |
| Statistical methods | 12 | (*a*) Describe all statistical methods, including those used to control for confounding  Differences in prevalence of infection among different groups were tested for statistical significance using the Chi-square test. The student’s t-test was used to determine the difference in mean age between males and females. |
|  |  | (*b*) Describe any methods used to examine subgroups and interactions  The prevalence of schistosomiasis-STH co-infections was also calculated. In this study individual schistosomiasis-STHs co-infection was considered for individuals who were infected with at least one schistosome species and at least one soil transmitted helminths species. District *S. haematobium* and *S. mansoni* co-endemicity was defined as the co-existence of *S. haematobium* and *S. mansoni* in the same implementation unit whether due to individuals being co-infected or due to different individuals being infected with different schistosome species. District schistosomiasis -STH co-endemicity was defined as the existence of at least a schistosome species and at least one STH species in the same district whether due to individuals being co-infected or due to different individuals being infected with different helminths.  The prevalence of heavy infection with any schistosome species was determined and stratified by gender and province. The prevalence of individuals heavily infected with any schistosome species was 5.8%. More males (7.1%) were heavily infected than females (4.7%), p = 0.001. The highest prevalence of heavy infection intensity with any schistosomes species was observed in Masvingo province (9.7%) followed by Midlands (9.6%) and Mashonaland Central province (9.1%), respectively. Of the 68 districts included in the national schistosomiasis and STH survey, 32 districts (50%) had prevalence of heavy infection with any schistosome species ≥ 5%. Seventeen districts (25%) had prevalence of heavy infection with any schistosome species ≥ 1% but < 5%. Four districts had the prevalence of heavy infection with any schistosome species >0% but < 1% and 15 (22.1%) districts had no detectable morbidity. |
|  |  | (*c*) Explain how missing data were addressed  Those with missing ages were allocated the average ages of the class as the group they were picked from according to the school register. In the analysis if parasitology data was missing the individual’s data was excluded from the analysis. |
|  |  | (*d*) If applicable, describe analytical methods taking account of sampling strategy  Differences in prevalence of infection among different groups were tested for statistical significance using the Chi-square test. The student’s t-test was used to determine the difference in mean age between males and females. Geographical positions of study schools were used to produce maps using geographical information system (GIS) software. MapInfo (Pitney Bowes Software Inc.) release 6.5 was used to process the data. A GIS data table was created for the schools using their geographic coordinates, which were converted into decimal format from their initial GPS reading format. |
|  |  | (*e*) Describe any sensitivity analyses  Data collected from the field was entered as egg counts while another variable was considered of either infected or uninfected by show of ova present and analysed. Differences in prevalence of infection among different groups were tested for statistical significance using the Chi-square test. The student’s t-test was used to determine the difference in mean age between males and females. The significance level was set at a p-value of 0.05. Geographical positions of study schools were used to produce maps using geographical information system (GIS) software. The GIS data codification and cleaning was carried out using Microsoft Excel. The clean and coded data were imported into a Microsoft Access database. Further analysis was conducted with Microsoft Access to integrate the different datasets into a single dataset. Then the integrated table was re-imported into Excel sheet before its conversion into the GIS data format. MapInfo (Pitney Bowes Software Inc.) release 6.5 was used to process the data. A GIS data table was created for the schools using their geographic coordinates, which were converted into decimal format from their initial GPS reading format. Out of 280 schools included in the national survey, 256 (91.4%) had geographic coordinates and were successfully integrated in the school GIS database. The prevalence class breaks are the recommended categorisation by World Health Organization. |
| Results | | |
| Participants | 13* | (a) Report numbers of individuals at each stage of study—eg numbers potentially eligible, examined for eligibility, confirmed eligible, included in the study, completing follow-up, and analysed  Reported below under (c) |
|  |  | (b) Give reasons for non-participation at each stage  None provision of urine and stool samples. Data was excluded from analysis of the individuals. While in few cases some GIS data was missing and the centres were later revisited and loged in. |
|  |  | (c) Consider use of a flow diagram  A total of 13 195 primary school children drawn from 280 schools were included in the study. Of these, 13067 (99.0%) had their age recorded. 12 464 (95.4%) and 6 (0.05%) were aged 6-9; 10-15 and >15 years old, respectively. Ages of 128 participants were not recorded. The mean age (standard deviation) of participants whose ages were recorded was 11.20 ± 1.39 years with males (11.33±1.45 years) being significantly older than females (11.07±1.31 years) (p < 0.0001). Thirteen thousand and thirty eight (98.8%) of the children examined were screened for *S. haematobium*, 12 249 (92.8%) were screened for *S. mansoni* and 12 252 (92.9%) were screened for STH. Not all participants were screened for all parasites because some could not provide stool or urine specimens on the day of sample collection or had their results missing before entry. |
| Descriptive data | 14* | (a) Give characteristics of study participants (eg demographic, clinical, social) and information on exposures and potential confounders  A total of 13 195 primary school children drawn from 280 schools were included in the study. Of these, 13067 (99.0%) had their age recorded. Five hundred and ninety seven [597 (4.6%)], 12 464 (95.4%) and 6 (0.05%) were aged 6-9; 10-15 and >15 years old, respectively. The mean age (standard deviation) of participants whose ages were recorded was 11.20 ± 1.39 years with males (11.33±1.45 years) being significantly older than females (11.07±1.31 years) (p < 0.0001). Thirteen thousand and thirty eight (98.8%) of the children examined were screened for *S. haematobium*, 12 249 (92.8%) were screened for *S. mansoni* and 12 252 (92.9%) were screened for STH. Not all participants were screened for all parasites because some could not provide stool or urine specimens on the day of sample collection or had their results missing before entry. Of the 280 schools included in the national survey, schistosomiasis was observed in 237 (84.6%) schools. In schools where schistosomiasis was detected, 116 (48.9%) had moderate prevalence (≥ 10 % but <50%) and 46 (19.5%) had high prevalence (≥ 50%). |
|  |  | (b) Indicate number of participants with missing data for each variable of interest  Only three districts (Gweru, Kwekwe and Bindura) out of 71 were left out due to limited resources. Ages of 128 participants were not recorded even though it is assumed that all participants were within the school systems within the same required age range. Not all participants were screened for all parasites because some could not provide stool or urine specimens on the day of sample collection or had their results missing before entry. |
| Outcome data | 15* | Report numbers of outcome events or summary measures  Of the 68 districts included in the national survey, the prevalence of schistosomiasis ≥ 50% was observed in 5 rural based districts. Prevalence of schistosomiasis ≥ 10% but < 50% was observed in 39 of the 68 districts (57.4%). Eighteen districts (26.5%) had the prevalence of schistosomiasis > 0% but < 10%. Seven rural and urban districts had prevalence of 0% whilst peri-urban areas around Harare and Bulawayo had an average schistosomiasis prevalence of 10.7% and 1.9% respectively. Eight districts had the prevalence of STH ≥ 15% whilst 35 (51.5%) districts had the prevalence of STH >0.0% but < 15%. Schistosomiasis was not detected in 9 of the 25 districts that were non-endemic to STH. The recommended Preventive Chemotherapy (PCT) strategies based on prevalence and schistosomiasis-STH co-infections according to WHO guidelines, annual mass drug administration for the control of schstosomiasiss was predicted in 5 districts whilst annual mass drug administration (MDA) for the control of STH was predicted in 7 districts. Biennial MDA for schistosomiasis control was predicted in 33 districts. Our results show that, of the 13195 participants screened for *S. mansoni* using both the Kato Katz and formol ether, 641 (5.3%) were diagnosed positive by the earlier technique and 672 (5.5%) were diagnosed positive by the later technique. However, after combining results obtained using the two techniques, 881 (7.2%) were diagnosed positive for *S. mansoni*. Contrary to the case of schistosomiasis, national distribution of STH was mapped for the first time in Zimbabwe through this study. The results showed that STH tends to occur in areas where schistosomiasis occurs, being common in the north, north eastern region, the eastern highlands and the southern region of Zimbabwe. |
| Main results | 16 | (*a*) Give unadjusted estimates and, if applicable, confounder-adjusted estimates and their precision (eg, 95% confidence interval). Make clear which confounders were adjusted for and why they were included |
|  |  | (*b*) Report category boundaries when continuous variables were categorized |
|  |  | (*c*) If relevant, consider translating estimates of relative risk into absolute risk for a meaningful time period  Of the 280 schools included in the national survey, schistosomiasis was observed in 237 (84.6%) schools. In schools where schistosomiasis was detected, 116 (48.9%) had moderate prevalence (≥ 10 % but <50%) and 46 (19.5%) had high prevalence (≥ 50%). Thirteen thousand and thirty eight (98.8%) of the children examined were screened for *S. haematobium*, 12 249 (92.8%) were screened for *S. mansoni* and 12 252 (92.9%) were screened for STH. Of the 12 252 participants screened for any STH, the overall combined prevalence of STH was 5.5%, ranging from 0% to 18.3% in provinces, 0% to 45% in districts and 0% to 78.7% in schools. There was no significant difference in prevalence of STH between males (7.5%) and females (6.9%), p = 0.231. The prevalence of STH was highest in Binga district (45.5%, 95%CI = 38.46-52.67) followed by Mutoko (43.5%, 95%CI = 35.55-51.72) and Murehwa district (40.6%, 95%CI = 34.07-47.46). There was significant variation in prevalence of STH between provinces, districts and schools (p < 0.0001). |
| Other analyses | 17 | Report other analyses done—eg analyses of subgroups and interactions, and sensitivity analyses  There was a significant variation in prevalence of schistosomiasis by settlement type: rural area (26.5%, 95%CI = 25.60-27.31); peri-urban area (9.9%, 95%CI = 8.04-12.09), urban-high density areas (8.9%, 95%CI = 7.52-10.37) and urban-low density areas (1.7%, 95%CI= 0.56-3.99), p < 0.0001. The overall prevalence of *S. haematobium* light and heavy infection intensity was 12.4% and 5.6% respectively. The overall prevalence of light, moderate and heavy infection intensity for *S. mansoni* was 3.6%; 1.4% and 0.3% respectively. There was a significant variation in prevalence of STH between settlement types (p <0.0001). The prevalence of STH was 6.5% (95%CI = 6.00-6.99) in rural areas; (1.7%, 95%CI = 0.92-2.81) in peri-urban areas; (1.6%, 95% = 1.00-2.40) urban high-density areas and (1.9, 95%CI = 0.50-4.67) urban-low density areas, p < 0.0001. The point prevalence of *A. lumbricoides* was scantly distributed in Zimbabwe and occurred in the north, north east and eastern highlands. *Trichuris trichiura* occurrence is insignificant in Zimbabwe. It was observed in few points in the North Eastern region and the Eastern Highlands of Zimbabwe. |
| Discussion | | |
| Key results | 18 | Summarise key results with reference to study objectives  Of the 68 districts included in the national survey, the prevalence of schistosomiasis ≥ 50% was observed in 5 rural based districts. Prevalence of schistosomiasis ≥ 10% but < 50% was observed in 39 of the 68 districts (57.4%). Eighteen districts (26.5%) had the prevalence of schistosomiasis > 0% but < 10%. Seven rural and urban districts had prevalence of 0% whilst peri-urban areas around Harare and Bulawayo had an average schistosomiasis prevalence of 10.7% and 1.9% respectively. Eight districts had the prevalence of STH ≥ 15% whilst 35 (51.5%) districts had the prevalence of STH >0.0% but < 15%. Schistosomiasis was not detected in 9 of the 25 districts that were non-endemic to STH. The overall arithmetic mean egg intensity (SE) for *S. haematobium* was 15.0 eggs/10ml (0.84), range (0-4 000 eggs/10ml) and that for *S. mansoni* was 7.65 epg (0.67), range (0-4 152 epg). The overall prevalence of *S. haematobium* light and heavy infection intensity was 12.4% and 5.6% respectively. The overall prevalence of light, moderate and heavy infection intensity for *S. mansoni* was 3.6%; 1.4% and 0.3% respectively. Of the 12 252 children screened for any STH, 9 828 (80.2%) lived in the rural areas, 830 (6.8%) lived in peri-urban areas, 1 380 (11.3%) lived in urban-high density areas and 214 (1.8%) lived in urban low-density areas. Schistosomiasis-STH co-endemicity was observed in 41 of the 68 districts (60.3%). |
| Limitations | 19 | Discuss limitations of the study, taking into account sources of potential bias or imprecision. Discuss both direction and magnitude of any potential bias  This study was cross sectional in nature and therefore could not provide empirical data on causality. Thus observations made can only be cautiously explained. The persistence of high prevalence of schistosomiasis throughout periods of all the 3 national surveys and current survey is not surprising as no control measures were instituted following the previous two surveys. Furthermore, the country went through serious social and environmental changes favouring transmission of schistosomiasis in the past two decades. However, the benefits of sporadic localized control interventions are evident in areas where pilot schistosomiasis control projects were implemented. |
| Interpretation | 20 | Give a cautious overall interpretation of results considering objectives, limitations, multiplicity of analyses, results from similar studies, and other relevant evidence  The overlap in distribution of schistosomiasis and STH in Zimbabwe may be explained by the overlap of conditions that favour co-existence of both schistosomiasis and STH. Putatively, these include existence of wet soils maintained by high rainfalls, optimal temperatures for parasites survival and livelihoods activities that pre-dispose communities to infections. The livelihoods activities like market gardening in the endemic areas, bathing and washing clothes in rivers increase the frequency of exposure of people to contaminated water and general poor sanitation facilities result in environmental contamination with faeces. The observed high prevalence of schistosomiasis in the rural areas compared to urban and peri-urban areas may be attributed to inadequate sanitary facilities, use of contaminated water sources for domestic chores that include washing clothes and bathing. It could also be due to risky (in terms of predisposition to schistosomiasis infection) livelihoods activities such as market gardening and brick moulding that are inherent in rural Zimbabwe. The situation in peri-urban areas is also worse than those in urban areas for some of the above stated reasons in particular poor sanitation and inadequate safe water for domestic use. Contrary to the case of schistosomiasis, national distribution of STH was mapped for the first time in Zimbabwe through this study. The results showed that STH tends to occur in areas where schistosomiasis occurs, being common in the north, north eastern region, the eastern highlands and the southern region of Zimbabwe. It should however be noted that examination of single urine and stool sample may have underestimated the prevalence and morbidity of infections. Thus, in order for Zimbabwe to achieve the set global goals: to eliminate schistosomiasis by 2020 and interrupt it by 2025 respectively, the country should implement uninterrupted PCT and complementary strategies including health education, improved sanitation and safe water supply as recommended by this study. |
| Generalisability | 21 | Discuss the generalisability (external validity) of the study results  The present study provides comprehensive baseline data showing geographic distribution of schistosomiasis and STH, their co-endemicity and the prevalence of heavy infection with any schistosome species in Zimbabwe. Intervention strategies intended to direct the country in controlling morbidity, eliminate and interrupt schistosomiasis and STH transmission are suggested. Uninterrupted annual MDA is recommended in districts where the prevalence of schistosomiasis and STH is ≥ 50% and ≥ 15% respectively. Similarly, annual MDA is recommended in districts where the prevalence of heavy infection by any schistosome species is ≥ 10%. Complementary strategies including health education, provision of safe water and adequate sanitary facilities should concurrently be implemented. The intervals for the co-administration of different medicines directed at different PCT neglected tropical diseases should be dependent on the NTD with the highest prevalence in any implementation unity. |
| Other information | | |
| Funding | 22 | Give the source of funding and the role of the funders for the present study and, if applicable, for the original study on which the present article is based  The study received funding from the Government of Zimbabwe, UNICEF, WHO and Helen Keller International. Drugs for treatment were received from Schistosomiasis Control Initiative. |

*Give information separately for exposed and unexposed groups.

**Note:** An Explanation and Elaboration article discusses each checklist item and gives methodological background and published examples of transparent reporting. The STROBE checklist is best used in conjunction with this article (freely available on the Web sites of PLoS Medicine at http://www.plosmedicine.org/, Annals of Internal Medicine at http://www.annals.org/, and Epidemiology at http://www.epidem.com/). Information on the STROBE Initiative is available at www.strobe-statement.org.
